# Comparison of the RNA Content of Extracellular Vesicles Derived from *Paracoccidioides* *brasiliensis* and *Paracoccidioides lutzii*

**DOI:** 10.3390/cells8070765

**Published:** 2019-07-23

**Authors:** Roberta Peres da Silva, Larissa G. V. Longo, Julia P. C. da Cunha, Tiago J. P. Sobreira, Marcio L. Rodrigues, Helisson Faoro, Samuel Goldenberg, Lysangela R. Alves, Rosana Puccia

**Affiliations:** 1 Departamento de Microbiologia, Imunologia e Parasitologia, Escola Paulista de Medicina-Universidade Federal de São Paulo (EPM-UNIFESP), São Paulo 04023-062, Brazil; 2 Laboratório Especial de Ciclo Celular—Center of Toxins, Immune Response and Cell Signaling-Center (CeTICS), Butantan Institute, São Paulo 05503-900, Brazil; 3 Bindley Bioscience Center, Purdue University, West Lafayette, IN 47907, USA; 4 Instituto Carlos Chagas—FIOCRUZ PR, Curitiba 81350-010, Brazil; 5 Instituto de Microbiologia da Universidade Federal do Rio de Janeiro, Rio de Janeiro 21941-590, Brazil

**Keywords:** *Paracoccidiodes*, extracellular vesicles, RNA-seq, mRNA, sRNA

## Abstract

*Paracoccidioides brasiliensis* and *P. lutzii* cause human paracoccidioidomycosis. We have previously characterized the <200-nt RNA sub-populations contained in fungal extracellular vesicles (EVs) from *P. brasiliensis* Pb18 and other pathogenic fungi. We have presently used the RNA-seq strategy to compare the <200- and >200-nt RNA fractions contained in EVs isolated from culture supernatants of *P. brasiliensis* Pb18, Pb3, and *P. lutzii* Pb01. Shared mRNA sequences were related to protein modification, translation, and DNA metabolism/biogenesis, while those related to transport and oxidation-reduction were exclusive to Pb01. The presence of functional full-length mRNAs was validated by in vitro translation. Among small non-coding (nc)RNA, 15 were common to all samples; small nucleolar (sno)RNAs were enriched in *P. brasiliensis* EVs, whereas for *P. lutzii* there were similar proportions of snoRNA, rRNA, and tRNA. Putative exonic sRNAs were highly abundant in Pb18 EVs. We also found sRNA sequences bearing incomplete microRNA structures mapping to exons. RNA-seq data suggest that extracellular fractions containing Pb18 EVs can modulate the transcriptome of murine monocyte-derived dendritic cells in a transwell system. Considering that sRNA classes are involved in transcription/translation modulation, our general results may indicate that differences in virulence among fungal isolates can be related to their distinct EV-RNA content.

## 1. Introduction

*Paracoccidioides brasiliensis* and *P. lutzii* cause human paracoccidioidomycosis (PCM), which is an endemic systemic mycosis prevalent in Latin American countries, but predominantly reported in Brazil [1]. The successful establishment of this fungal infection depends on the thermo-dependent dimorphic transition from environmental conidia to the yeast phase in the lung alveoli. *P. lutzii* isolates are responsible for the majority of PCM cases in the Central and Northern Brazil and are represented by the highly studied Pb01 isolate [2]. *P. brasiliensis* is a complex of several phylogenetic groups originating from Brazilian regions and other countries [3,4]. The Pb18 and Pb3 isolates represent the phylogenetic main species S1 and a cryptic PS2 group, respectively [5]. PS2 isolates cause a milder form of murine PCM when compared to S1 isolates, apparently by stimulating a predominant Th1-type of cellular immune response [6], but the fungal features that contribute to their distinct virulence are not known. A broad analysis of genome diversity in *Paracoccidioides* showed that the phenotypic differences among phylogenetic groups and species are not due to differences in large protein families, but probably due to unique genes with no orthologs in other lineages [7].

Extracellular vesicles (EVs) constitute an important cellular mechanism of non-conventional secretion across all kingdoms. EV is a general term used to define spherical bi-layered-membrane structures that group, according with their origin, (a) exosomes of 30–100 nm deriving from multivesicular bodies; (b) microvesicles or ectosomes of 100–1000 nm originating from either budding or invagination of the plasma membrane; (c) apoptotic bodies that are larger than 1000 nm [8,9,10,11,12]. EVs can safely transport to the extracellular environment and distant sites a vast number of proteins, including active enzymes and toxins, lipoproteins, DNA, RNA, polysaccharides, and pigments [9]. EVs are involved in a broad array of intercellular communication and active biomolecule transfer that have implications in physiological cellular processes, infection, and disease [13,14]. EVs from microorganisms can directly interact with cells of the immune system and affect the infection process [15]. Distinct RNA specimens can be transported inside EV-like structures or associated with RNA-binding proteins and high-density lipoprotein complexes; therefore, the standardization of EV isolation and RNA purification is essential step to evaluate those populations [12,16]. After the detection of mRNA and small RNAs (sRNA) within EVs [17], the role of micro RNA (miRNA) from EVs in the genetic regulation of recipient cells has been widely addressed [18,19]. EVs also contain different classes of long non-coding RNA (lncRNAs) that are potentially able to regulate the transcription by recruiting epigenetic modifiers in the recipient cells [20].

We have previously characterized the sRNA fraction contained in EVs isolated from *P. brasiliensis* (isolate Pb18), *Cryptococcus neoformans*, *Candida albicans*, and also from *Saccharomyces cerevisiae* [21]. The sRNA fraction included sequences of various sizes shorter than 250 nt, specifically, 114 small non-coding (nc)RNA sequences of the small nucleolar (sno)RNA and transporter (t)RNA classes. We also identified 1246 conserved miRNA-like sequences, from which 20 were common to all samples and 47 had differentially represented levels. There was a small percentage of mRNA (10%) that co-fractionated in the <200 nt-enriched fraction and was also characterized. Interestingly, these sequences were unique to EVs from each species [21].

The present work aimed at comparing the RNA populations carried by EVs from two isolates (Pb18 and Pb3) representative of *P. brasiliensis* lineages S1 and PS2 and *P. lutzii* (Pb01), since differences in RNA content could be valuable to partially explain differences in virulence among isolates. We also tested the functionality of EVs mRNA and if extracellular fractions containing fungal EVs can evoke transcriptional changes in dendritic cells.

## 2. Materials and Methods

### 2.1. Fungal Growth Conditions

For EVs isolation from culture supernatant, *P. brasiliensis* isolates Pb18, Pb3, and *P. lutzii* Pb01 were cultivated for 2 days at 37 °C, under shaking, in defined Ham’s F12 medium supplemented with 1.5% glucose (500 mL), as previously described [21].

### 2.2. EVs Isolation

*Paracoccidioides* EV preparations were performed as described [21,22]. In brief, culture supernatants from two 500-mL cultures were centrifuged for 15 min at 4000× *g*, then for 30 min at 15,000× *g*, followed by filtration through a 45 μm membrane and concentration using Amicon ultrafiltration membranes (100-kDa cutoff). The cell-free, debris-free concentrated supernatant was ultracentrifuged at 100,000× *g* for 1 h at 4 °C and the pellets containing EVs were washed in phosphate-buffered saline (PBS). This protocol for EV isolation results in EVs with size peaks between 40 to 80 nm (suggestive of exosomes), as verified by nanoparticle-tracking analysis (NTA) for Pb18; small peaks over 150 nm are also visible. The final pellets were lyophilized for RNA extraction.

### 2.3. Murine Monocyte-Derived CD11c+ Cells (MoDC)

The cells extracted from the C57BL/6 mice bone marrow were cultured in 6-well plates (2.5 × 10^5^ cells/well) in 2 mL of complete DMEM high glucose (Gibco) supplemented with 10% FBS (fetal bovine serum, Gibco), glutamine (2 mM) (Gibco), 2-mercaptoethanol (0.05 mM) (Gibco), penicillin/streptomycin (100 U/mL; 100 μg/mL) (Gibco), and 20 ng/mL GM-CSF (Gibco). The plates were kept at 37 °C at 5% CO_2_ and, after four days of incubation, 1 mL of media was replaced by 1 mL of fresh DMEM supplemented with 40 ng/mL GM-CSF. By the seventh day, 500 µL of fresh medium without GM-CSF replaced the same volume of old medium. On day 9, non-adherent cells and loosely adherent cells were harvested, stained in ice-cold PBS containing 1% BSA using anti-CD11c monoclonal antibody-PerCP-Cyanine5.5 (eBioscience) and sorted by FACS.

### 2.4. Indirect Co-Culture of Murine Monocyte-Derived CD11c+ Cells (MoDC) and Pb18

Indirect co-culture of MoDC with Pb18 was performed using a transwell system (0.4-μm membrane porosity) in 1 mL DMEM supplemented with 10% FBS in each chamber. Pb18 yeast cells (1 × 10^6^) were seeded in the upper chamber and 1 × 10^6^ CD11c+ cells (dendritic cells, MoDC) were placed in the lower chamber of a six-well plate. The plates were incubated for 48 h at 37 °C, at 5% CO_2_, the MoDCs were harvested, washed and used for RNA extraction.

### 2.5. RNA Isolation and Sequencing

Total RNA extraction from *Paracoccidioides* EVs and fractionation were carried out as described [21]. Small and large RNA were fractionated using the miRNeasy mini kit (Qiagen, Germantown, MD, USA) and the RNeasy MinElute Cleanup Kit (Qiagen), according to the manufacturer’s protocol. We used an Agilent 2100 Bioanalyzer (Agilent Technologies, Santa Clara, CA, USA) to test the integrity of the RNA preparations before sequencing. Total RNA was extracted from 1 × 10^6^ MoDC cells using the RNeasy kit (Qiagen). The RNA was eluted in RNAse-free water and stored at −70 °C. The RNA-seq was carried out as previously described [21].

### 2.6. Data Analysis

The RNA sequences were analyzed by CLC Genomics Workbench© v 7.0 (Qiagen), using both the corresponding *Paracoccidioides* genomes from NCBI as reference (Pb18-ABKI02000000, Pb3-ABHV02000000, and Pb01-ABKH02000000) and the *Saccharomyces cerevisiae* non-coding database (https://www.yeastgenome.org/). All the sequencing analyses were performed in triplicate, except for the < 200nt enriched-fraction from Pb18 EV that had a duplicate evaluated. The parameters used for the alignments were: mismatch cost (2), insertion cost (3), deletion cost (3), length fraction (0.8), and similarity fraction (0.8). The abundance values are presented in reads per kilobase of exon model per million mapped reads (RPKM) [23]. For the analysis of secondary structure, the RNA-seq reads obtained for the < 200 nt fraction were directly evaluated in a PPFold plugin in the CLC Genomics Workbench v.10.1, using default parameters [24]. The sequences were up to 50-nt long.

### 2.7. Data Access

The RNA-seq data have been deposited at the Sequence Read Archive (SRA) database under the accession number (SRA: SRP022849).

### 2.8. In Vitro Translation and Protein Analysis

The in vitro translation assay was performed according to the manufacturer’s instructions. Briefly, 0.6 µg total RNA from Pb18 EVs were incubated for 90 min at 30 °C with 35 µL Rabbit Reticulocyte Lysate (Promega) containing leucine/methionine (0.5 pM) and 40 U RNAseOUT (Invitrogen). The reaction product was filtered through a Millipore^®^ membrane (0.45 µm), diluted 5 times in PBS-NaCl 0.3 M and incubated at 4 °C with 200 µL Ni-NTA (Amersham) until the resin became red due to hemoglobin binding [25]. The suspension was centrifuged (5 min at 16,000× *g* at 4 °C) and the proteins were precipitated overnight (−20 °C) from the supernatant by addition of 3 volumes of cold acetone. The sample was spun down for 30 min at 16,000× *g* (4 °C) and washed with cold acetone. The precipitated proteins were denatured, reduced, trypsinized, and desalted as described [25]. The tryptic peptides were suspended in 0.1% formic acid (FA) and loaded to an LTQ-VelosOrbitrap (Thermo Fisher Scientific, Waltham, MA, USA) through a coupled nanoHPLC (Proxeon, Odense, Denmark). The samples were desalted and concentrated in a pre-column (10-μm C18 beads, Phenomenex, 100 μm × 2 cm, Torrance, CA, USA) and separated at 200 nL/min in a reverse-phase capillary column (5-μm beads, Phenomenex 10 cm × 75 mm). The peptides were eluted by a linear gradient from 5% to 40% of solvent A [solvent A: 5% acetonitrile (ACN)/0.1% FA; solvent B: 100% ACN/0.1% FA] over 90 min and for an additional 15 min with up to 95% of solvent B. The eluted peptides were directly injected in the mass spectrometer via a nanoelectrospray set at 2.2 kV. All analyses were performed in the positive ionization mode at the 50–2000 *m*/*z* range. The mass spectrometer was operated in the data-dependent acquisition mode to automatically switch between one orbitrap full-scan and ten ion trap tandem mass spectra. The *.raw data files were processed at MaxQuant 1.3.0.5 and the searches performed at Andromeda against a merged database of *P. brasiliensis* Pb18 (https://www.ncbi.nlm.nih.gov/genome/?term=txid502780[Organism:noexp]) and *Oryctolagus cuniculus* of TrEMBL (downloaded at 2015/02) using a 1% false discovery rate (FDR). The search parameters included: (i) carbamidomethylation of cysteine residues as a fixed modification; (ii) oxidation of methionine residues as a variable modification; and (iii) 6 ppm and 0.5 Da for MS1 and MS2 tolerance, respectively. Proteins identified as contaminants at a reverse database and “only identified by site” were excluded from further analysis.

## 3. Results

We characterized the ncRNA and mRNA sub-populations contained in EVs isolated from culture supernatant samples of the representative *Paracoccidioides* isolates Pb18, Pb3, and Pb01. Our results compile the analysis of three independent biological replicates for each fungal sample. In addition, we evaluated the effect of Pb18 extracellular fractions containing EVs on the transcription pattern of a murine monocyte-derived (CD11c+) cell line co-cultivated with Pb18.

### 3.1. Paracoccidioides EVs Carry Functional mRNA

In a previous study, we characterized mRNA sequences in fungal EVs that were co-purified in the < 200-nt RNA fraction [21]. We presently analyzed large RNA fractions to identify the most abundant mRNA sequences contained in *Paracoccidioides* EVs. The reads obtained from the mRNA libraries (>200 nt) were aligned with each isolate-specific genome at NCBI (Pb18-ABKI02000000, Pb3-ABHV02000000, and Pb01-ABKH02000000). For data validation, we only considered sequences with expression values of RPKM > 100 in all biological replicates. Those sequences were individually accessed and only mRNA transcripts with reads covering at least 50% of the CDS were validated (Table 1).

**Figure 1:**
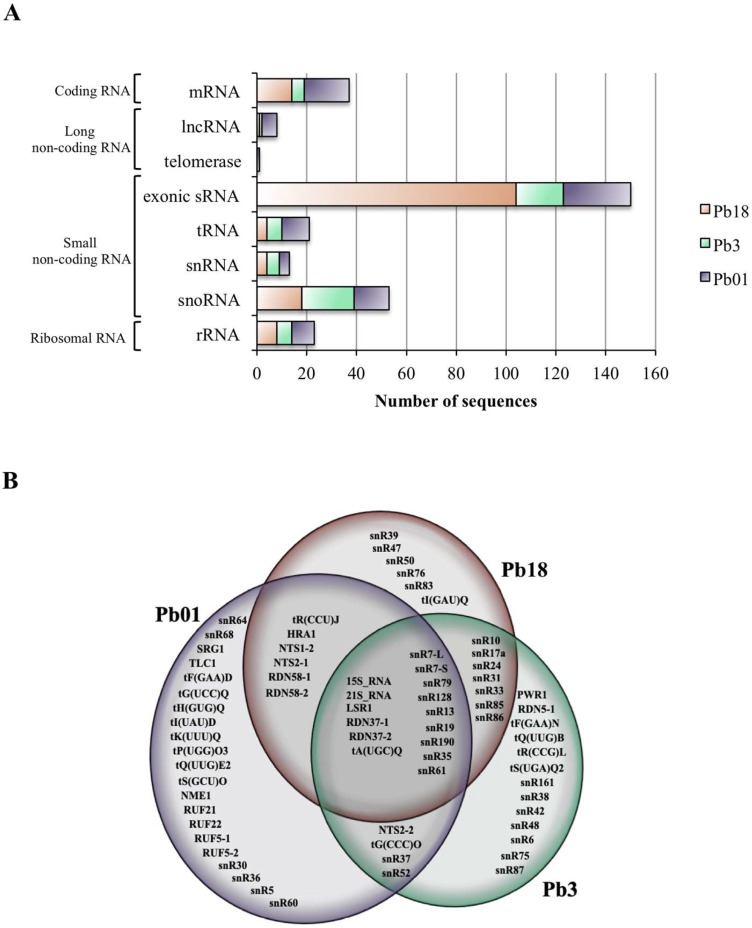
(**A**) Graph showing the number of sequences in each class of RNA identified in EV preparations from Pb18, Pb3 and Pb01. (**B)** Venn diagram showing all ncRNA sequences found in EV preparations from Pb18, Pb3 and Pb01.

According to the aforementioned parameters, we validated a total of 30 mRNA sequences in EV samples from Pb18 (14), Pb3 (5), and Pb01 (18), as seen in Table 1 and Figure 1A. The sequences coding for polyubiquitin and histone H3 were found in EVs from all isolates, while those coding for calmodulin, elongation factor 1-alpha, and histone H2a were detected in two isolates (Table 1). The remaining sequences were exclusive to either Pb18 or Pb01. For Pb3, there was only one exclusive mRNA coding for a pre-mRNA splicing factor. In general, the mRNA sequences were mainly related to protein modification, translation, and DNA metabolism/biogenesis (Table 1). The latter function grouped most of the overlapping transcripts among the three samples. On the other hand, sequences related to transport and oxidation-reduction were only detected in Pb01. Twenty-seven out of the 30 validated sequences had over 69% of CDS coverage. Considering that we only validated mRNAs with over 50% coverage of each CDS, comparisons with our previous work were unfruitful because the validation parameters were distinct [21].

To confirm the presence of functional full-length mRNA in *Paracoccidioides* EVs, we carried out an in vitro translation assay. The Pb18 mRNA EV sample was translated using a rabbit reticulocyte lysate. Thereafter, the contaminating hemoglobin was removed by incubation with NI-NTA agarose and the remaining translated proteins were analyzed by LC-MS/MS. We found five proteins that were translated from EV mRNA (Table 2). Among them, only the transcript for a heat shock protein (PADG_07715) was included in Table 1 (RPKM > 100). Together, our results show that the *Paracoccidiodes* EVs contain functional full-length mRNAs and that they are differentially represented in Pb18, Pb3, and Pb01.

### 3.2. sRNA Sequences Aligning to mRNA Exons (Exonic sRNA)

The EV RNA reads detected in the < 200-nt fraction were initially aligned with each *Paracoccidioides* isolate-specific genome at NCBI. We noticed that this fraction was composed mostly of short 25-nt sequences in average that aligned to a specific region of a particular mRNA exon. In our previous work we observed that over 80% of the reads found in the <200-nt RNA fraction from EVs mapped to exons not only in *P. brasiliensis* Pb18, but also in *C. albicans* and *S. cerevisiae*, however not in *C. neoformans* [21]. In fungi, exonic short interfering RNAs (ex-siRNA) have been described to match to unique exon sites in either the reverse or the forward directions. They originate directly from single-strand RNA via an RNA-dependent RNA polymerase to generate double-stranded RNA (dsRNA) and are then converted to interfering RNA by Dicer [26]. Therefore, we presently deepened our investigation of the reads that had high depth of coverage in exonic regions. Each high-coverage coding sequence was manually accessed to evaluate the alignment position of the reads. Considering the reads generated by the sequencing of both < and > 200-nt fractions, 160 mRNA sequences showed high values of depth of coverage. Among them, only 30 had reads covering more than 50% of the entire mRNA, as detailed earlier. The remaining 130 were represented by small sequences that aligned at unique sites. We therefore called them exonic sRNA. We found a total of 104 (Pb18), 19 (Pb3), and 27 (Pb01) exonic sRNA in EVs that corresponded to a specific exonic region (5′, 3′or middle) of a single mRNA (Table 3 and Figure 1A). For Pb18 EVs, 53% of those sequences were only in the forward orientation (F), while 68% and 56% were only in the reverse (R) direction in Pb3 and Pb01 EVs, respectively.

Four EV exonic sRNA sequences mapped to a common transcript in all *Paracoccidioides* isolates: calcium calmodulin-dependent protein kinase (PADG_07652), nucleotide binding (PADG_03535), transcription factor tfiiib complex subunit brf1 (PADG_00916), and a hypothetical protein (PADG_11439). As seen in Table 3, most of the exonic sRNAs were specific to EVs from Pb18 (89) and Pb01 (21), whereas only four were exclusive to Pb3 EVs. Ten fragments mapped to homologous transcripts in Pb18 and Pb3 EVs. Most of the target sequences have unknown functions, while the others are related to carbohydrate/protein/DNA metabolism, translation, oxidation-reduction, and the signaling process (Table 3).

In conclusion, although the number of putative exonic sRNA was 4 to 5-fold higher in Pb18 EVs, 10 target sequences seemed to be characteristic of *P. brasiliensis*, whereas 21 were exclusive to *P. lutzii*. These results reveal a previously unknown diversity in the composition of fungal EVs at the genus level.

### 3.3. Comparison of EV ncRNA Classes in Paracoccidioides EVs

The different classes of ncRNA contained in Pb18, Pb3, and Pb01 EVs were analyzed by aligning the <200-nt reads with the ncRNA database from the *Saccharomyces* Genome Database. The results revealed the presence of 71 different sequences of ncRNA in *Paracoccidioides* EVs, from which 15 were common to all isolates and 17 were shared by two of them (Figure 1B). The most abundant class of ncRNA found in *Paracoccidiodes* EVs was the small nucleolar (sno)RNAs (33), followed by tRNAs (16), rRNAs (10), long ncRNAs (7), and small nuclear (sn)RNAs (4), as seen in Figure 1A. The snoRNA and tRNA were also the most abundant ncRNA populations described in our previous work not only for *P. brasiliensis*, but also for *C. albicans*, *C. neoformans*, and *S. cerevisiae* [21].

The number of total ncRNA detected in EVs varied with the isolate, being slightly more abundant in *P. lutzii* Pb01 (45) than in *P. brasiliensis* Pb3 (39) or Pb18 (35). The class profiles also differed between species. For *P. brasiliensis* EVs, snoRNA represented about 55% of the total, followed by rRNA (23% in Pb18 and 15% in Pb3), and 11 to 15% tRNA and snRNA (Figure 1A). For *P. lutzii* EVs, we found similar proportions (20 to 31%) of snoRNA, rRNA, and tRNA, followed by 9 to 13% of snRNA and other ncRNAs (Figure 1A).

### 3.4. Secondary Structure in the EVs.

**Figure 2:**
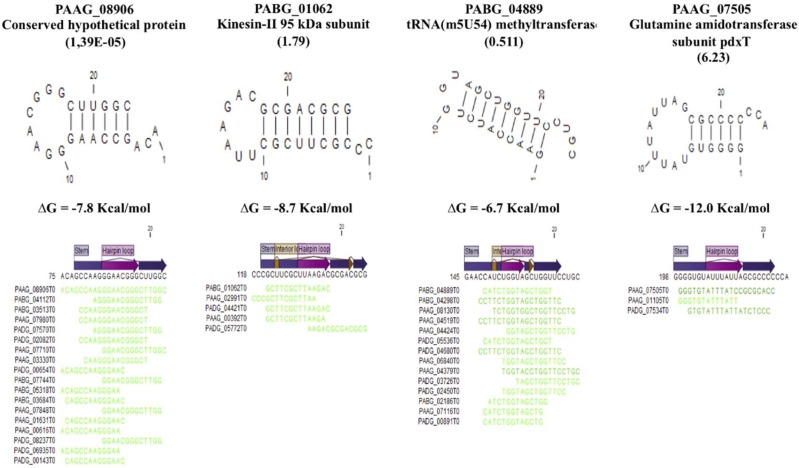
Secondary structures with the lowest deltaG among 42 sRNA sequences with values below −3 Kcal/mol. The Blast search result for these sequences is shown.

In our previous publication [21], we identified putative miRNA-like (milRNA) sequences in fungal EVs that aligned with mature miRNA following a search in the miRNA database of all organisms (http://www.mirbase.org). In the present work, we searched for sRNA sequences (up to 50 nt) bearing secondary structure in order to have a more precise view of the presence of milRNA in *Paracoccidioides* EVs. We applied the secondary structure analysis to RNA fragments detected in EVs at high abundance (RPKM > 5000) and for further characterization we selected 42 RNA sequences bearing secondary structures with free energy values below −3.0 Kcal/mol. The sequences with the most negative values are shown in Figure 2. We performed an alignment with these 42 sequences to infer their function and identify in which strand they would align (Supplemental Table S1). Two were present in all EV samples, while 23 were found in both Pb3 and Pb01 EVs. Interestingly, 50% of the structured RNAs aligned at the complementary strand of the RNA, thus suggesting that they could act as miRNA or the transcripts (Supplemental Table S1). Most of the structured RNAs localize to transcripts that code for hypothetical proteins, however we could also identify RNAs that align to transcripts of kinesin-II, glutamine amidotransferase and lysine methyltransferase (Figure 2).

### 3.5. Paracoccidioides EVs Might Modulate the Transcriptome of Dendritic Cells

Dendritic cells make the bridge between the innate and adaptative immune responses, which ultimately define the course of the fungal infections [27]. The disease progression in paracoccidioidomycosis depends on dominant Th1 or Th2 types of immune response. The Th1-driven inflammatory immune response is responsible for protection against the disease and the IL-12 expressed by dendritic cells is detrimental to stimulate this type of response [28]. In this context, we investigated if extracellular fractions containing EVs produced by *P. brasiliensis* Pb18 would affect gene expression in recipient dendritic cells. In order to do that, we characterized the transcriptome of murine monocyte-derived CD11c^+^ (MoDC) cells co-cultivated for 48 h with the Pb18 yeast cells in a transwell system. We compared the sequences with those of the controls cultivated in the absence of fungal cells. In the transwell system, we had the fungal (upper compartment) and MoDC cell (bottom compartment) cultures communicating by a porous membrane that allowed the transit to the bottom well of soluble molecules and EVs up to 0.4 µm in size.

**Figure 3:**
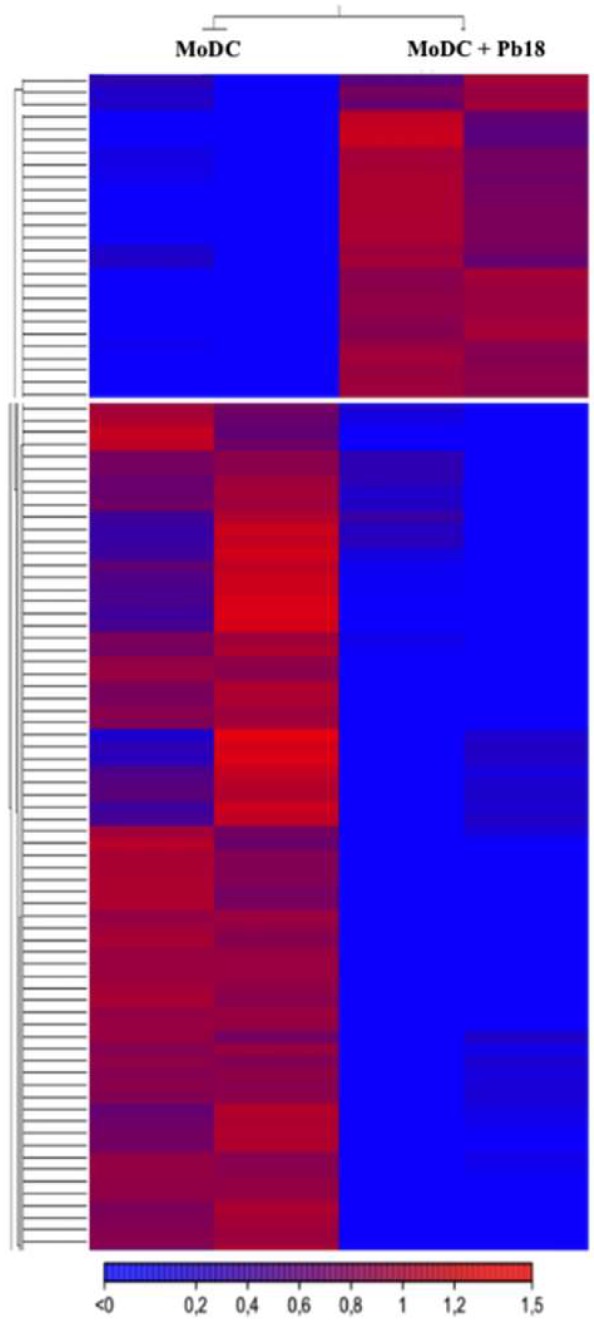
Heat map of transcripts from MoDC cells that were modulated over 2-fold and had a *p*-value < 0.05 when co-cultivated indirectly with Pb18 in a transwell system.

We observed that the indirect co-culture with Pb18 led to a slight alteration in the levels of mRNA expressed by MoDC cells when compared to the control (Figure 3 and Table S2). We detected 20 upregulated and 28 downregulated mRNAs (Figure 3). We chose to follow highly stringent criteria in this analysis (similar levels of expression in both replicates, FDR ≤ 5% and log FC ≥ 2), considering we had duplicates, but not triplicates. Among the upregulated transcripts, three code for membrane proteins (Ankar, Unc13c, and Smim7) and two code for mitochondrial proteins associated to translation elongation (mt-Te and mt-Tv) (Table S2). Among the downregulated transcripts, there was 25% enrichment for transcripts related to gene expression regulation (transcription and translation). When we applied the hypergeometric test to the annotated transcripts for the gene set enrichment analysis, the *p*-value of the gene expression group was 2.40 × 10^5^, thus reinforcing the significance of this enrichment (Table S2). It was interesting to notice that three transcripts that code for transcription factors (Gabpb2, Pknox1, and Zfp575) are among the downregulated MoDC mRNAs in indirect co-cultures with Pb18 (Table S2), suggesting that sRNAs from the fungal EVs were potentially able to modulate the gene expression of the recipient cell. It is important to note that we have preliminary cytometry data showing that Pb18 yeast cells can actively release EVs under our transwell experimental conditions and that MoDC cells can internalize fungal EVs, as depicted in Figure S1.

## 4. Discussion

In the present work we have shown that the features of mRNA and sRNA sub-populations contained in EVs from *P. brasiliensis* Pb18, Pb3, and *P. lutzii* Pb01 differ considerably between species and also between representative isolates of the same species. The results suggest that *Paracoccicioides* extracellular fractions containing EVs can modulate the transcription profile of dendritic cells. By using in vitro translation, we also reported for the first time in pathogenic fungi that the EV mRNAs are active and can be translated.

The mRNA sequences presently detected in the >200-nt RNA fraction of *Paracoccidoides* EVs varied in number with the isolate and only five orthologs were common to EVs from more than one isolate. We have recently compared the characteristics of mRNA sequences from EVs exported by two *H. capsulatum* isolates, specifically G186AR and G217B [29]. The latter isolate lacks cell wall alpha-1,3-glucan, which is a virulence factor [30]. The number of EV mRNA sequences varied from 93 in G186AR (mostly related to metabolic processes) to 31 in G217B (related to transport pathways possibly requiring vesicles, oxidation-reduction, and translation mechanisms). In the present analysis for *P. brasiliensis*, which is genetically related to *Histoplasma* [31], transcripts associated with DNA metabolism/biogenesis and protein modification were enriched in all samples, while those related with translation and transport prevailed, respectively, in Pb18 and Pb01.

While there are several pieces of evidence suggesting the role of fungal EVs on host cells, specially by interfering with the course of the immune response [32,33,34,35,36], it has recently been demonstrated that EV-like liposomal particles can cross the fungal cell wall inwards [37], thus opening the possibility that EVs can be uptaken and signal fungal cells. It has recently been demonstrated that EV-associated plant sRNA can silence virulence genes in a fungal pathogen, which agrees with the hypothesis that EVs mediate trans-kingdom regulation of gene expression [38]. On the other hand, Bielska et al. (2018) demonstrated that EVs produced by virulent *Cryptococcus gattii* were essential to signal quiescent strains within phagolysosomes located in distant body sites, which then became virulent; that phenomenon was apparently mediated by EV protein and RNA [39]. We have here detected active mRNA within *Paracoccidioides* EVs and we can envision that they might be uptaken by other neighboring or distant fungal cells and play a role in the host-fungal relationship by delivering virulence transcripts. Table 1 includes, for e.g., heat shock proteins like Hsp 70 (PAAG_08003, in Pb01 EVs) and Hsp 90-like (PADG_07715, in Pb18 EVs) that have a role in virulence of dimorphic fungi [40].

On the other hand, among the siRNA described in fungi, the exonic ex-siRNAs promote gene silencing in *Mucor circinelloides and Fusarium graminearum* [26,41,42]. In these species, the ex-siRNAs correspond to sense and antisense siRNAs converted into double-stranded RNA (dsRNA) via RNA-dependent RNA polymerase and Dicer. They regulate translation of the same mRNA that originated it. The presence of sense and antisense exonic sRNA-like in *Paracoccidioides* EVs suggests that these molecules could be involved in gene silencing via dsRNA. In the present work, we can point out the finding of exonic sRNA-like sequences, in Pb18 EVs, that map to α-amylase (PADG_04422), which is essential to the synthesis of the virulence factor α-glucan [30,43], and β-glucanase (PADG_04922), that cleaves dectin-1 ligand β-glucan present in the cell wall. We propose that they could have a role in regulating the expression of virulence genes in other fungal cells.

The comparison of the small ncRNA subtypes in *Paracoccidioides* EVs showed clear inter-species diversity. In *P. lutzii*, the percentage of snoRNA was about half that in *P. brasiliensis*, whereas for tRNA and other ncRNA it was, respectively, 2- and 4-fold higher. Consequently, 20 sequences were exclusive of *P. lutzii* EVs (mostly tRNA) versus six for Pb18 and thirteen for Pb3. In our experimental conditions, *H. capsulatum* EVs transport almost exclusively rRNA and tRNA [29]. The tRNA sequences prevailed in the EVs from isolate G186AR and most of them were not detected in EVs from the G217R strain. The fragments of tRNAs (the tRFs) are implicated in diverse processes in the cell, from regulation of cell viability, protein synthesis, apoptosis, to RNA metabolism, including turnover and stability [44]. The tRFs present in the EVs form *Trypanosoma cruzi*, the causing agent of Chagas disease, can be transferred to other parasites and promote cell communication and/or to host cells to modulate gene expression or facilitate infection [45].

The gene-silencing mechanism known as RNA interference (RNAi) is prompted by small noncoding (s)RNAs averaging 25 nt that act at either a post-transcriptional or post-translational level. The RNAi-related sRNAs are short interfering RNAs (siRNA), microRNAs (miRNA), and piwi-interacting RNAs (piRNA). In fungi, the conventional miRNA pathway has only been demonstrated in *C. neoformans* [46], while alternative miRNA-like (mil-RNA) pathways have been reported in, for ex., *Fusarium oxysporum* [47], *Penicillium marneffei* [48], and *Neurospora crassa* [49].

We have previously detected 145 sequences in EVs from *P. brasiliensis* Pb18 that matched those of mature miRNA deposited in the miRNA database (mirbase). More recently, Curcio et al. [50] searched for matches of mil-RNAs already described in fungi, discarded those located in genes and looked for pre-miRNA secondary structure in the genome by considering their flanking nucleotides. They found that 11 mil-RNAs previously reported in fungal EVs matched the criteria for mil-RNA in the *P. brasiliensis* Pb18 genome. Besides, the authors found that in Pb18 the paralogous genes for Argonaute (1 and 2) and Dicer (1 and 2) (PADG_00716; PADG_03108; PADG_11946; PADG_07189) seem to be induced in the pathogenic yeast phase of the fungus, suggesting that mechanisms involving RNAi can be functional in this species [50]. We have presently shown partial secondary RNA structures in sRNA sequences from EVs that was differentially represented in the *Paracoccidioides* isolates. These sequences aligned to exons, notably in the reverse position. We found similar results for *H. capsulatum* [29], but the role of these molecules has to be investigated further. It was interesting to find, in *Histoplasma* EVs, a series of RNA-binding proteins and one of them, Snd1, is a component of the RNA-induced silencing complex (RISC) that is part of the RNAi machinery [51]. In Pb18 EVs [52] we have not found RNA-binding proteins that matched those found in *Histoplasma* EVs (data not shown).

Modulation of the host immune system by EV components has been described in *C. neoformans*, *C. albicans*, *Malassezia sympodialis*, *P. brasiliensis* and *Sporothrix brasiliensis*. The fungal EVs activate immune cells in vitro inducing the release of pro-inflammatory mediators, suggesting a role of fungal EVs in activating the immune system to respond to the fungal infection [32,33,34,35,36]. Importantly, in all studies performed so far, it was uncertain if the density of EVs used to stimulate host cells corresponded to that observed during physiologic and/or pathogenic conditions. Therefore, we opted for a transwell-based experimental system where the amount of fungal EVs corresponded to that physiologically produced by *P. brasiliensis*, even with the limitation that molecules not related to EVs may participate in the interaction with host cells. We observed that the Pknox1 and Gbpb2 transcription factors were highly downregulated (65-fold and 30-fold, respectively) in MoDC cells upon indirect co-coculture with *P. brasiliensis* in the transwell experiment. Pknox1 (Pbx/knotted 1 homeobox) belongs to the HOX family of transcription factors and is critical for the immune system homeostasis as it regulates the expression of IL-10 in macrophages and dendritic cells [53]. Gbpb2 (GA-binding protein subunit beta 2) is critical for T cell development and the expression regulation of IL-7R alpha [54]. IL-7 is associated with improved immune response against bacterial and viral infections, and also in fungal sepsis [55] and *Pneumocystis* infection [56]; however, a direct correlation of IL-7 and paracoccidioidomycosis has not been evaluated yet. On the other hand, IL-10 is a potent anti-inflammatory cytokine that is generally associated with increased infection in paracoccidioidomycosis [57]. Our experimental design using a transwell system did not allow distinguishing between the biological effects of EVs from other secreted molecules. However, considering our data suggesting that *Paracoccidioides* Pb18 produce EVs that are uptaken by MoDC in a transwell system (Figure S1), we feel entitled to assume that EVs from *P. brasiliensis* Pb18 might help to modulate the MoDC response, possibly favoring the infection at the early stages of interaction with cells of the innate immune system. This modulation could be related to regulatory sRNA carried by the fungal EVs or even by surface ligands like DC-SIGN mannose ligands exposed in the EV surface [58].

Considering that sRNA classes are involved in transcription/translation modulation in a variety of systems and potentially also in *Paracoccidioides*, our general results may indicate that differences in virulence among fungal isolates could be related to their distinct EV RNA content. That hypothesis will hopefully be experimentally tested.

## Figures and Tables

**Table 1:** Proteins corresponding to validated mRNA sequences (RPKM > 100) found in *Paracoccidioides* EVs. The sequences are grouped according with their functions. Coverage used for validation is indicated, as well as the gene access codes for each isolate.

Pb18 Feature ID	Pb3 Feature ID	Pb01 Feature ID	Sequence Description	Coverage	GO
**Protein Modification**					
		PAAG_08003	hsp70-like protein	100%	ion binding
		PAAG_05980	ubiquitin-conjugating enzyme e2-16 kda	80%	ligase activity
PADG_07715			hsp90-like protein	70%	protein folding, response to stress
PADG_01605	PABG_03078	PAAG_07080	polyubiquitin	70%	protein modification process
PADG_11111			nuclear transport factor 2	75%	protein targeting
		PAAG_05679	ATP-dependent molecular chaperone hsc82	90%	nucleic acid binding transcription factor activity, protein folding
**Carbohydrate Metabolism**					
PADG_02145			glycogen phosphorylase	70%	carbohydrate metabolic process
**Translation**					
PADG_05025			60s ribosomal protein l26	70%	translation
PADG_00692		PAAG_11418	elongation factor 1-alpha	100%	translation
PADG_03326			40s ribosomal protein s9	80%	translation
	PABG_05744		pre-mrna splicing factor	70%	translation
**Oxidation-Reduction**					
		PAAG_03216	thiol-specific antioxidant	90%	oxidoreductase activity
**Transport**					
		PAAG_11262	hsp7-like protein	100% + MR	transmembrane transport
		PAAG_03058	high-affinity methionine permease	100% + 5′R	transmembrane transport
		PAAG_07634	gtp-binding protein rhoa	70%	vesicle-mediated transport
**DNA Metabolism or Biogenesis**					
PADG_00873	PABG_02444	PAAG_07099	histone h3	100%	chromosome organization
	PABG_05588	PAAG_08917	histone h2a	90%	chromosome organization
		PAAG_08918	histone h2b	70%	chromosome organization
PADG_06568			tctp family protein	50%	cell differentiation
	PABG_03449	PAAG_08247	calmodulin	70%	ion binding
**Other/Unknown Function**					
PADG_02280			hypothetical protein	75%	Unknown
PADG_02399			calcium-binding protein	70%	Unknown
PADG_04049			hypothetical protein	60%	Unknown
PADG_08402			hypothetical protein	100%	Unknown
PADG_12385			ser thr protein phosphatase family protein	60%	Unknown
		PAAG_00340	conserved hypothetical protein	85%	unknown
		PAAG_12435	hypothetical protein	80%	unknown
		PAAG_12692	ATP synthase subunit beta	95% reverse	unknown
		PAAG_12694	plant senescence-associated protein	100% reverse	unknown
		PAAG_02087	kelch-like protein 38	70%	unknown

**Table 2:** Protein sequences detected by in vitro translation of total mRNA extracted from Pb18 EVs. The mean RPKM corresponds to that found in the RNA-seq analysis.

Feature ID	Name	RPKM Mean
PADG_00648	conserved hypothetical protein	7
PADG_04056	14-3-3 protein epsilon	25
PADG_04810	GTP-binding nuclear protein GSP1/Ran	37
PADG_06159	sulfate transporter	12
PADG_07715	Hsp90-like protein	127

**Table 3:** Target mRNA of exonic sRNA sequences found in EVs from Pb18, Pb3, and Pb01 isolates. The position of sRNA in the exon is indicated as 5′, 3′, and M (middle), as well as the direction (F, forward; R, reverse). The sequences are grouped according with their functions.

Pb18 Feature ID	Alignment	Pb3 Feature ID	Alignment	Pb01 Feature ID	Alignment	Sequence Description	GO
**Protein modification**							
PADG_01365	5′R/3′F					disulfide isomerase	protein folding
PADG_03114	MR					phospho-2-dehydro-3-deoxyheptonate aldolase	amino acid metabolic process
PADG_04092	3′F					peptidyl-prolyl cis-trans isomerase b	protein modification process
PADG_05011	MF					peptidyl-prolyl cis-trans isomerase-like 3	protein modification process
PADG_05560	3′F					26s proteasome regulatory subunit rpn-1	small molecule metabolic process
PADG_05731	3′F					hypothetical protein	amino acid metabolic process
PADG_07241	5′F					dihydroxy-acid dehydratase	amino acid metabolic process
PADG_07550	MR	PABG_04093	MR			microsomal signal peptidase subunit	peptidase activity
				PAAG_05962	5′F	proteasome regulatory particle subunit	catabolic process
**Carbohydrate Metabolism**							
PADG_02145	5′F	PABG_06801	5′F			glycogen phosphorylase	carbohydrate metabolic process
PADG_03169	MR					alpha-glucan synthase ags2	carbohydrate metabolic process
PADG_04432	MF					alpha-amylase	carbohydrate metabolic process
PADG_04922	3′F					cell wall glucanase	carbohydrate metabolic process
PADG_05870	5′R/5′F					glucan synthesis regulatory protein	carbohydrate metabolic process
**Lipid Metabolism**							
PADG_12430	5′F	PABG_07295	3′F			amp-binding	long-chain fatty acid metabolic process
**Oxidation-Reduction**							
PADG_04419	3′R	PABG_01064	3′R			proline oxidase	catabolic process
				PAAG_05378	MF	d-3-phosphoglycerate dehydrogenase	oxidoreductase activity
				PAAG_01937	3′F	duf887 domain-containing protein	biosynthetic process
				PAAG_11262	mRNA/MR	hsp7-like protein	transmembrane transpor
PADG_05080	5′F					pyridoxamine phosphate oxidase family protein	ion binding
PADG_06181	3′F					c-5 sterol desaturase	lipid metabolic process
PADG_07431	5′R/3′F/3R					chloroperoxidase-like protein	homeostatic process
PADG_12214	5′R					alcohol dehydrogenase	oxidoreductase activity
**Translation**							
PADG_00995	MF					ubiquitin-40s ribosomal protein s27a	translation
PADG_02452	MF					rna polymerase rpb1 c-terminal repeat domain-containing protein	
PADG_02484	MF					valyl-trna synthetase	tRNA metabolic process
PADG_05025	MF					60s ribosomal protein l26	translation
PADG_06082	3′F					pre-mrna splicing factor	translation
PADG_06160	MF					eukaryotic translation initiation factor 2 alpha subunit	translation
PADG_06191	5′F	PABG_06964	3′R			trna isopentenyltransferase	tRNA metabolic process
PADG_06522	5′F					glycine--trna ligase	
PADG_06833	5′F					ATP-dependent rna helicase drs1	ribosome biogenesis
PADG_08605	5′F					40s ribosomal protein s28	translation
PADG_01891	MF					translation initiation factor rli1	translation
PADG_02317	3′R					translation machinery-associated protein 17	
**Signaling Process**							
PADG_05447	3′F					vacuolar membrane-associated protein iml1	signal transduction
PADG_06642	5′F					ste ste7 protein kinase	response to stress
PADG_08337*	3′F					gtp-binding protein rhoa	signal transduction
**Transport**							
PADG_00326	MF					adp-ribosylation factor-like protein 1	vesicle-mediated transport
PADG_01303	3′F					abc transporter	biosynthetic process
PADG_01567	3′R					sorting nexin 3	vesicle-mediated transport
PADG_05084	5′R					high affinity copper transporter	transmembrane transport
PADG_05821	5′F					importin	
PADG_06982	3′F					ncs1 family nucleobase:cation symporter-1	transmembrane transport
PADG_08101	3′F					mrna cleavage factor complex component pcf11	transport
PADG_03535	MR	PABG_01859	MR	PAAG_01288	MR	nucleotide binding	ion binding, ligase activity
		PABG_11660	3′F			endoplasmic reticulum vesicle protein 25	vesicle-mediated transport
				PAAG_03058	mRNA/5′R	high-affinity methionine permease	transmembrane transport
				PAAG_03479*	3′R	mfs multidrug	transmembrane transport
				PAAG_11682	3′R	duf1903-domain-containing protein	transport
				PAAG_12134	3′F	mfs drug transporter	transmembrane transport
**DNA Metabolism or Biogenesis**							
PADG_00916	MR	PABG_02494	5′R	PAAG_07153	5′R	transcription factor tfiiib complex subunit brf1	biosynthetic process
PADG_03251	3′F					c6 finger domain protein acr-	biosynthetic process
PADG_05475	5′R					dna-directed rna polymerases and iii 145 kda polypeptide	biosynthetic process
PADG_06799	MR					camk camkl kin4 protein kinase	cell division
PADG_07652*	MR	PABG_06307*	MR	PAAG_05737*	MR	calcium calmodulin-dependent protein kinase	cytoskeleton organization, cell division
PADG_11268	3′F					tyrosine recombinase -like	DNA metabolic process
PADG_11500	MF					fungal specific transcription	biosynthetic process
PADG_12343	3′F					serine threonine protein kinase	regulation of transcription
		PABG_00984	5′F	PAAG_03968	5′F	c6 transcription	biosynthetic process
		PABG_03356	3′R			homeobox transcription	biosynthetic process
**Other/Unknown Function**							
PADG_00069	3′R					hypothetical protein	
PADG_00138	5′R					hypothetical protein	
PADG_00639	5′R/5′F					hypothetical protein	
PADG_01127	5′R					rna-binding protein	ion binding
PADG_01198	3′F					vps9 domain	
PADG_01219	MF/MR					hypothetical protein	
PADG_01476	3′F			PAAG_06944	3′F	c2h2 finger domain	ion binding
PADG_01739	3′R						
PADG_01808	MF					hypothetical protein	
PADG_01880	3′F					u-box domain-containing protein	
PADG_01892	3′R					formin binding protein	
PADG_02119	MR	PABG_03545	MR			protein	
PADG_02181	5′R					had superfamily	isomerase activity
PADG_02764	5′F					disulfide bond formation protein d	
PADG_02871	3′R					cfem domain-containing protein	
PADG_02926	3′R					tam domain methyltransferase	
PADG_03103	3′R					phytase	phosphatase activity
PADG_03162	3′R					domain protein	
PADG_03436	MF					3 exoribonuclease family protein	
PADG_03788	MF					polyadenylation factor subunit 64	
PADG_04049	5′R/5′F					hypothetical protein	
PADG_04157	5′R					cellobiose dehydrogenase	
PADG_04417	3′F					hypothetical protein	
PADG_04448	5′F					polarized growth protein	
PADG_04473	MR					duf647 domain-containing protein	
PADG_04629	3′R					protein	
PADG_04760	5′F					multiple myeloma tumor-associated protein 2 like	
PADG_04828	MF/MR					adenylosuccinate lyase	biosynthetic process
PADG_05226	MF					protein	
PADG_05352	5′R					ubiquitin carboxyl-terminal hydrolase 19	
PADG_05378	5′F					protein	
PADG_05589	MR					protein	
PADG_05603	3′R					increased rdna silencing protein 4	ion binding
PADG_06044	3′F					ankyrin repeat containing protein	
PADG_06240	3′F					hypothetical protein	
PADG_06449	3′R					phosphotransferase enzyme family protein	
PADG_07205	5′F					protein	
PADG_07675	MF/MR					cellular morphogenesis protein	
PADG_07897	5′R/5′F/5′NS					hypothetical protein	
PADG_07988	3′F/3′R					conserved hypothetical portein	
PADG_07990	MF					tam domain methyltransferase	
PADG_08617	3′F/3′ R	PABG_07734	3′R			hypothetical protein	
PADG_11034	5′R/5′F	PABG_11827	5′R/5′F			protein	
PADG_11035	3′F					protein	
PADG_11277	5′R					protein	
PADG_11439	5′R	PABG_00126	5′R	PAAG_11926	5′R	hypothetical protein	
PADG_11473	5′R					hypothetical protein	
PADG_11562	MF					hypothetical protein	
PADG_11613	5′R					hypothetical protein	
PADG_11652	3′R	PABG_01675	3′R			kh domain rna binding protein	RNA binding
PADG_11758	3′R/3′F/3′NS					hypothetical protein	
PADG_11762	MF					hypothetical protein	
PADG_12001	3′F					hypothetical protein	
PADG_12385	5′R/5′F	PABG_06506	3′R			ser thr protein phosphatase family protein	
		PABG_06943	3′R			hypothetical protein	
		PABG_12403	MR/MF/MNS			hypothetical protein	
				PAAG_01376	5′R	hypothetical protein	
				PAAG_01424	5′F/MF	iron-sulfur cluster assembly accessory protein	biosynthetic process
				PAAG_01967	MR	hypothetical protein	
				PAAG_03361	MF	predicted protein	
				PAAG_04613	MR	vacuolar protein sorting-associated protein 51	
				PAAG_05089	3′F		
				PAAG_07600	3′F		
				PAAG_07877	3′F	protein	
				PAAG_11750	5′F/5′R	hypothetical protein	
				PAAG_12291	3′R	camp-dependent protein kinase pathway protein	kinase activity
				PAAG_12405	MR	hypothetical protein	
				PAAG_12534	5′R	mitochondrial 37s ribosomal protein nam9	rRNA binding
				PAAG_12681	MR	hypothetical protein	ion binding

## Supplementary Materials

The following files are available online, Figure S1: Uptake of EVs from Pb18 by MoDC cells in a transwell system, Table S1: List of genes from Pb18, Pb3, and Pb01 matching partially structured sRNA, as indicated. Separated lists contain those found exclusively in one isolate or shared by two or three of them, Table S2: MoDC genes differentially expressed (upregulated in green and downregulated in pink) after incubation with Pb18 cells in a transwell system, as analyzed using the RNA-seq strategy.

## Abbreviations

EVs	Extracellular vesicles
GM-CSF	Granulocyte macrophage colony-stimulating factor
miRNA	Micro RNA
ncRNA	Non-coding RNA
PCM	paracoccidioidomycosis
sRNA	Small RNA
snoRNAs	Small nucleolar RNA
tRNA	Transporter RNA
