# Supplementary material for: Comparison of the RNA Content of Extracellular Vesicles Derived from Paracoccidioides brasiliensis and Paracoccidioides lutzii
Source: Cells. 2019 Jul 23;8(7):765. doi: 10.3390/cells8070765 (PMC6678485; doi:10.3390/cells8070765)
Supplement: Supplementary file 1 [file cells-08-00765-s001.zip › Legend Figure Supple1 Peres da Silva.docx]

**Figure S1: Uptake of EVs from Pb18 by MoDC cells in a transwell system.** Pb18 yeast cells were washed three times in sterile PBS and disaggregated by passing samples through narrow-gauge syringes. We used the fluorescent lipid dye PKH67 (Sigma, according to the manufacturer's protocol) to label the equivalent of one preparation of Pb18 EVs and Pb18 yeast cells (1x10^7^), which were metabolic active for EV production or heat killed at 65^o^C for 1 h (negative control for EV production). Briefly, the EVs and fungal cells were suspended in 0.5 ml of Diluent C (Sigma) and PHK67 was added to a final concentration of 2x10^-6^ M. After 5-min incubation, 1 ml of BSA 1% was added, the samples were pellet by centrifugation and washed 3 times with PBS. The MoDC were obtained as described in Materials and Methods.  Co-culture assays were performed in six-well plates containing a transwell with a 0.4-μm-pore size, as shown in the schematic representation on the left. A total of 1x10^6^ MoDC were plated in the bottom of the wells and 1x10^6^ yeast cells were deposited on the top compartment of the transwell membrane. Incubation was carried out in high-glucose DMEM medium supplemented with 10% BFS (bovine fetal serum, Life Technologies). To evaluate if the EVs would pass through the well membrane and be uptaken by MoDC,  we evaluated a control where 10 μM of EVs (lipid content) was added to the medium containing yeast cells. After 24, 48 and 72h of incubation at 36°C at 5% CO_2_, the content of the upper chamber was discarded, the MoDC were washed in PBS and double labeled with antibodies to CD11c and MHC class II. For blocking non-specific interactions of Fc-receptors, MoDC were incubated for 30 min at 4° C with B57/Bl6 (1:30) pre-immune serum. After another series of washes, the MoDC were incubated with PerCP/Cy5.5 anti-mouse CD11 followed by PE/Cy7 anti-mouse MHC class II. The cells were washed, fixed in 1% paraformaldehyde and evaluated by flow cytometry to verify the uptake of fungal EVs by dendritic cells.

The figure on the right shows that after 24 h of incubation, 10% of MoDCs had internalized EVs released by Pb18 yeast cells and this percentage reached 24% in the presence of 10 μM of Pb18 isolated EVs. After 48 and 72 h, these values reached approximately 60% (in the absence of EVs) and 85% (in the presence of EVs). The results suggest that Pb18 yeast cells actively released membrane EVs under our experimental conditions, since co-cultivation of MoDC with heat-killed Pb18 resulted in only 2% positive cells for PKH67. The results also suggest that Pb18 EVs (both actively released and added) were uptaken by MoDC. When Pb18 EVs were added directly to MoDC cultures and evaluated after 24 h of incubation, 93% of the MoDC cells were positive (not shown), suggesting that part of the EVs were retained by the transwell system. The data shown in Figure S1 have been considered preliminary because they are the result of a single experiment.
