# Supplementary figures and images for: Comparison of the RNA Content of Extracellular Vesicles Derived from Paracoccidioides brasiliensis and Paracoccidioides lutzii
Source: Cells. 2019 Jul 23;8(7):765. doi: 10.3390/cells8070765 (PMC6678485; doi:10.3390/cells8070765)

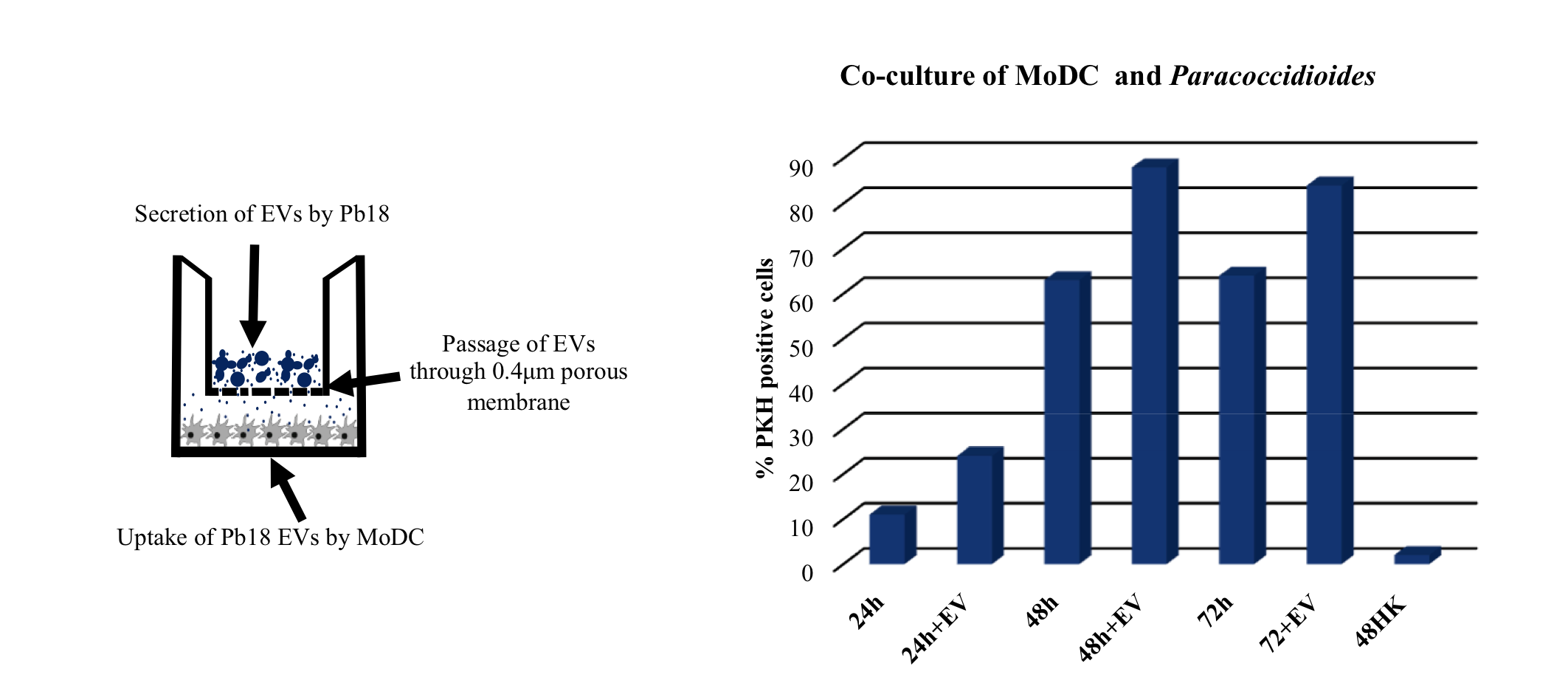

Supplement: Supplementary file 1 [file cells-08-00765-s001.zip › Figure1Suptiff.tiff]
